# ﻿*Leucheriapeteroana* (Nassauvieae, Asteraceae), a new species of *Leucheria* endemic to the Andes of Central Chile, and insights into the systematics of Nassauviae

**DOI:** 10.3897/phytokeys.248.133202

**Published:** 2024-11-07

**Authors:** Nicolás Lavandero, Fernanda Pérez, Nicolás Pinilla

**Affiliations:** 1 Facultad de Ciencias Biológicas, Pontificia Universidad Católica de Chile, Avenida Libertador B. O'Higgins 340, Santiago, Chile Pontificia Universidad Católica de Chile Santiago Chile

**Keywords:** Andes, Asteraceae, Laguna Teno, *Leucheria*, Maule, Nassauvieae, taxonomy

## Abstract

A new species, *Leucheriapeteroana***sp. nov.**, endemic to a restricted area of the Andes of Central Chile, is here described. Using newly sequenced nDNA and cpDNA data, the phylogenetic affinities of *Leucheria* and closely related taxa within Nassauvieae are revisited. This new species shows a unique set of characters that clearly distinguish it from other species of *Leucheria*. Phylogenetic analyses place this perennial species close to annual species found in the pre-Andean environments of Central Chile. A detailed description, distribution map, insights about its habitat, conservation status, and photographs are provided.

## ﻿Introduction

The genus *Leucheria* Lag. is one of the largest genera within the Nassauvieae tribe. It occurs in the Southern Cone of South America, distributed across Peru, Bolivia, Chile, and Argentina, including the Falkland Islands (Crisci 1976; Katinas et al. 2022). Most species are found within the Patagonian-Andean and Subantarctic Phytogeographic domains (Cabrera and Willink 1973). *Leucheria* has a rich and complex taxonomic history. For a complete and detailed history of its taxonomy, see Crisci (1976). In the latter work, the first modern revision of the genus, 46 species were recognized. Since Crisci (1976), at least three new species (Katinas et al. 2008a; Katinas et al. 2018; Lavandero et al. 2020) and a new variety (Ratto et al. 2014), which was later elevated to species level (Jara-Arancio et al. 2019), have been described. A recent work by Apodaca et al. (2021) made a significant reclassification, synonymizing 10 annual species of *Leucheria* into *Leucheriatomentosa* (Less.) Crisci. In the most recent taxonomic synopsis by Katinas et al. (2022), the number of species of *Leucheria* was reduced to 29. More recently, Muñoz-Schick and Moreira (2022) included *Leucheriagraui* Katinas, M. C. Tellería, & Crisci within the synonymy of *Leucheriaapiifolia* Phil., further reducing the current number of accepted species of *Leucheria* to 28.

The diversity center of *Leucheria* overlaps with the Central and Southern Chilean biodiversity hotspot (Moreira-Muñoz et al. 2012), one of 35 world biodiversity hotspots recognized by Myers et al. (2000) and later by Mittermeier et al. (2005), due to their great diversity and high levels of endemism, combined with a past and ongoing loss of habitat and biodiversity (Myers et al. 2000). Central Chile features a Mediterranean-type climate, although significant climatic heterogeneity exists due to latitudinal and altitudinal gradients, ranging from sea-level to up to 6570 m (Armesto et al. 2007; Luebert and Pliscoff 2017). The extraordinary environmental heterogeneity in this region, along with fluctuating changes due to the glacial history throughout the Quaternary, may have led to the higher species diversity and endemism observed in this area (Arroyo et al. 1995; Villagrán 1995).

Phylogenetic relationships within Nassauvieae have been the subject of several studies, from Phenetics (Crisci 1974) to Phylogenomics (Zhang et al. 2024). In particular, the relationship of *Leucheria* to *Oxyphyllum* and *Marticorenia* was earlier suggested by Crisci (1980). Hellwig (1985) suggested a close relationship of *Leucheria* to *Moscharia*, later questioned by Katinas (1994). Early molecular studies, based on the *ndhF* gene of the chloroplast genome, suggested a close relationship to *Jungia* L.f. (Kim et al. 2002). Subsequently, Panero and Funk (2008), using 10 chloroplast loci, found *Leucheria* as sister to a clade formed by *Nassauvia*, *Perezia*, *Acourtia*, *Dolichlasium*, *Trixis* and *Jungia*. However, Panero and Funk (2008) only sampled 9 genera within Nassauvieae. A broader sampling was used by Katinas et al. (2008c) where *Leucheria* is placed as sister to *Polyachyrus*, and this clade appears as sister to *Moscharia*, using both nuclear ITS and the chloroplast *trnL-trnF* intergenic spacer. Luebert et al. (2009) examined the relationships of monotypic *Oxyphyllum* within Mutisieae. By using the plastid *rbcL* and *ndhF* genes, the *trnL-trnF* spacer and nuclear ITS region, their work supported the close relationship of *Oxyphyllum* to *Leucheria* and *Polyachyrus*. More recently, Jara-Arancio et al. (2017), based on a broader sampling within Nassauvieae, and using the ITS region and two chloroplast intergenic spacers, placed *Leucheria* as sister to a clade formed by *Moscharia* and *Marticorenia*. More recent studies, using target capture methods (Mandel et al. 2019) and phylotranscriptomics (Zhang et al. 2021; Zhang et al. 2024), have only used a limited sampling within the tribe. Until now, generic and infrageneric relationships within Nassauvieae remain unresolved, with at least two genera, *Criscia* and *Cephalopappus*, without any genetic information available.

In the context of a modern taxonomic revision of the genus, unusual specimens of *Leucheria* were collected in the vicinity of the Lagunas del Teno (35°11'15"S, 70°33'33"W), in the Andes of the Maule Region of Chile. This area is characterized by a steep topography, with elevations up to 4112 m above sea level (Volcán Azufre). This area of the Andes Mountain range is characterized by active volcanic activity, with six volcanic complexes actively monitored in the present (SERNAGEOMIN 2018). Although several botanical collections have been made around this area, most were done near roads, which are limited to a few Andean Mountain passes and international border crossings between Chile and Argentina.

This work aims to describe a new species of *Leucheria*, providing a distribution map as well as information on its habitat, ecology and phenology. A provisional assessment of its conservation status is also provided. We further investigated its phylogenetic affinities, aiming to re-evaluate the phylogeny of *Leucheria* presented by Jara-Arancio et al. (2017), and the systematics and taxonomy of the most recent taxonomic synopsis of the genus by Katinas et al. (2022).

## ﻿Methods

### ﻿Herbarium and fieldwork

**Figure 1.**
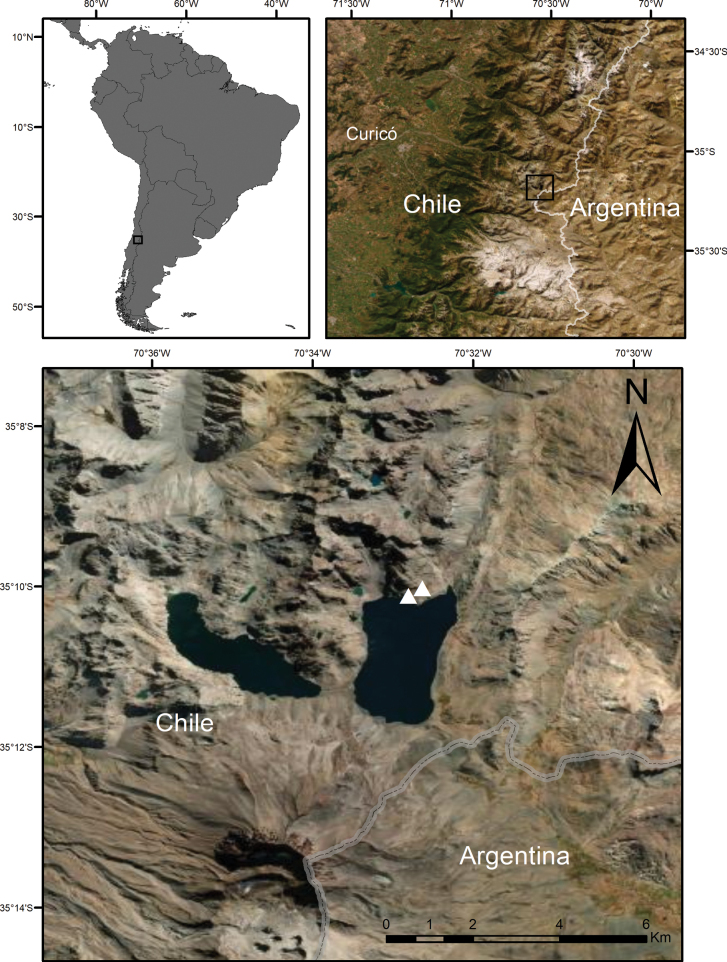
Distribution map of *Leucheriapeteroana* (white triangles) in Chile, Maule Region, based on the type locality and collections.

In March 2019, a botanical exploration was conducted around the Lagunas de Teno (2549 m). Specifically, we collected around the Laguna El Planchón (2549 m a.s.l.), one of the two lakes forming the Lagunas del Teno (Fig. 1). This lake is of glacial origin, created by the natural damming of the glaciers due to the activity of the Volcanic complex Planchón-Peteroa (Caputo et al. 2013). Specimens of *Leucheria* that could not be assigned to any of the currently accepted species were found. Herbarium specimens were collected, together with leaf material preserved in silica gel and capitula preserved in 70% ethanol. Herbarium specimens were deposited at SGO herbarium. A systematic examination of herbarium specimens of *Leucheria* found at CONC and SGO was carried out. The descriptions and keys were prepared after examining all available specimens.

### ﻿Conservation status

A tentative assessment of the conservation status of the species was made using the International Union for Conservation of Nature (IUCN 2012) categories and criteria, following the most recent guidelines (IUCN Standards and Petitions Committee 2024). The extent of occurrence (EOO) and area of occupancy (AOO) were calculated using GeoCat (Bachman et al. 2011).

### ﻿Taxon sampling and phylogenetic analysis

DNA sequences for nDNA (ITS), as well as cpDNA intergenic spacers (*rpl32-trnL* and *trnF-trnL*) were obtained from GenBank (www.ncbi.nlm.nih.gov/Genbank) for the outgroup genera within Mutisioideae and Barnadesioideae, including 25 of the 27 currently accepted genera of Nassauvieae (Katinas et al. 2008b). Due to conflicts in the identification of the herbarium samples used by Jara-Arancio et al. (2017), we decided to redo the phylogenetic analysis. We collected field samples for most of the taxa recognized by Crisci (1976) and subsequently by Katinas et al. (2022), which can be considered a “lumper” perspective of the former. All sequences of *Leucheria*, except for *Leucheriacantillanensis* Lavandero (Lavandero et al. 2020), were generated in the present study.

Total genomic DNA was extracted from silica-dried material collected in the field using the Qiagen DNeasy Plant Mini Kit (QIAGEN, Santiago, Chile) following the manufacturer’s instructions. Genomic DNA was used to amplify by PCR one nuclear region, the internal transcribed spacer region (ITS), and the chloroplast intergenic spacers *trnL-trnF* (Taberlet et al. 1991) and *rpl32-trnL* (Shaw et al. 2007). For the ITS region, we used the newly generated primers ITS4_leu (5’ TGATATGCTTAAACTCAGCGGG 3’) and ITS5_leu (5’ GGAAGGAGAAGTCGTAACAAGG 3’), modified from White et al. (1990). For the *trnL-trnF* region, we used the c and f primers following Taberlet et al. (1991). For the *rpl32-trnL* region, we used the primers described in Shaw et al. (2007). We amplified all regions of *Leucheria* in 25 μl PCR reactions using the following thermocycling conditions: initial denaturation of 95 °C for 5 min; 35 cycles at 95 °C for 1 min, a specific annealing temperature for 1 min (51 °C for *trnL-trnF* and *rpl32-trnL*; 55 °C for ITS), 72 °C for 1 min; and a final elongation period of 72 °C for 15 min. Sanger sequencing was performed in the Plataformas UC de Secuenciación y Tecnologías Ómicas, Pontificia Universidad Católica de Chile, using the ABI PRISM 3500 xl Genetic Analyzer (Applied Biosystems™). GenBank accession numbers for all DNA sequences used in this study are given in Suppl. material 3.

The assembled sequences were aligned using the MAFFT v7.450 algorithm (Katoh et al. 2002; Katoh and Standley 2013) in Geneious Prime 2022.2.1 (https://www.geneious.com). Phylogenetic analyses were run for both Maximum-likelihood (ML) (Felsenstein 1981), using RAxML-AVX3 version (Stamatakis 2014) included in RAxMLGUI v.2.0 beta (Silvestro and Michalak 2012; Edler et al. 2020), and Bayesian inference (BI) using MrBayes x64 v3.2.7 (Ronquist et al. 2012), respectively. The best-supported model of nucleotide sequence evolution for each partition was determined based on the Akaike Information Criterion (AIC) using MrModeltest v2 (Nylander 2004). For both partitions, the GTR+I+G model was selected. Before analysing the combined nuclear and chloroplast regions, an Incongruence Length Difference (ILD) Test was performed in PAUP v. 4.0a (Swofford 2003). Phylogenetic reconstruction was performed for each region independently, to compare topological incongruences between the nuclear and chloroplast dataset. For the combined analysis, two partitions were used, corresponding to the nuclear and the chloroplast regions. Maximum likelihood analyses were run using the GTRGAMMA approximation, including the proportion of invariant sites (+I option). The analysis included 1000 ML slow bootstrap replicates with 100 runs. Bayesian analyses were conducted under the respective best fit models for each partition, with two independent runs of 15 million generations each, sampling every 10000 generations. Time series plots and effective sample size (ESS) were analysed using TRACER v.1.7 (Rambaut et al. 2018) to check convergence for each run. The first 3 million generations were discarded as burn-in.

## ﻿Results

### ﻿Molecular phylogenetic analyses

The total DNA alignment contained 2841 characters (718 ITS, 950 *trnL-trnF*, and 1173 *rpl32-trnL*) representing 34 ingroup and 40 outgroup accessions. The incongruence-length difference test showed a significant conflict between the nuclear and chloroplastidial partitions (P < 0.001), so the combined dataset analysis must be interpreted cautiously, considering the topological discordances in some of the clades. In both ML and BI analyses (Suppl. materials 1, 2), there is a consistent incongruence between nuclear and chloroplast data regarding the position of Mutisieae (represented by *Mutisiaspinosa* and *Adenocaulonchilense* in this work). Nuclear data places this tribe as sister to a clade formed by Onoserideae and Nassauvieae, whereas chloroplast data places this tribe within Nassauvieae, and Onoserideae (represented by *Plaziadaphnoides* and *Gypothamniumpinifolium* in this work) as sister to this clade. However, all analyses and datasets retrieved *Macrachaenium*, placed within Nassauvieae by Panero and Funk (2008), as sister to *Mutisia*. Within Nassauvieae, Spinolivailicifoliasubsp.ilicifolia appears as sister to the whole clade for the nuclear dataset, whereas its placement is unresolved for the chloroplast dataset. A consistent clade is retrieved by both datasets consisting of *Berylsimpsonia*, *Trixis*, *Jungia*, *Pleocarphus*, *Ameghinoa*, *Dolichlasium* and *Leunisia* (PP = 1.0, BS = 100 for ITS; PP = 0.95, BS = 77 for chloroplast). Another clade consistently retrieved (PP = 1.0, BS = 87 for ITS; PP = 0.98, BS = 73 for chloroplast) is formed by *Nassauvia*, *Triptilion*, *Calopappus*, *Pamphalea*, *Perezia*, *Calorezia*, *Acourtia*, *Burkartia*, and *Holocheilus*. Within this clade, both datasets consistently retrieved a clade formed by *Acourtia*, *Burkartia* and *Holocheilus* (PP = 1.0, BS = 100 for ITS; PP = 0.96, BS = 88 for chloroplast), and another clade formed by *Nassauvia*, *Triptilion*, *Calopappus*, *Pamphalea*, *Perezia*, and *Calorezia* (PP = 1.0, BS = 99 for ITS; PP = 1.0, BS = 47 for chloroplast). The position of *Holocheilus* differs in the chloroplast ML analysis, as it appears in the latter clade.

**Figure 2.**
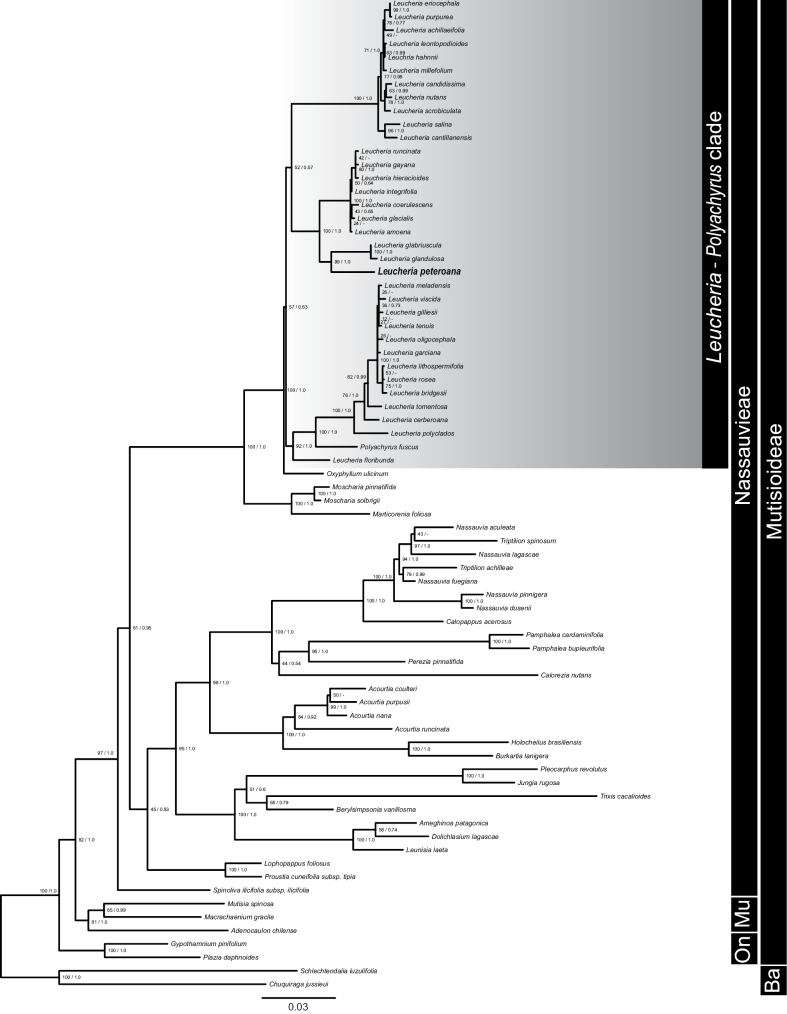
Phylogeny of Nassauvieae resulting from Maximum likelihood analysis of the combined nuclear ITS and plastid *rpl32-trnL* and *trnL-trnF* dataset. For each node, the values of bootstrap support under Maximum likelihood and Bayesian posterior probabilities are to the left and right of the slash, respectively. The new species, *Leucheriapeteroana* is highlighted in bold. Ba: Barnadesioideae, On: Onoserideae, Mu: Mutisieae.

In both datasets, a well-supported clade formed by *Marticorenia*foliosa, *Moscharia*, *Oxyphyllum*, *Polyachyrus* and *Leucheria* was retrieved (PP = 1.0, BS = 100 for both ITS and Chloroplast). In both datasets, *Marticorenia* appears as sister to *Moscharia* (PP = 1.0, BS = 100), and this clade is sister to *Oxyphyllum*, *Polyachyrus* and *Leucheria*. *Oxyphyllum* appears as sister to *Leucheria* and *Polyachyrus* in the nuclear dataset with moderate support (PP = 0.98, BS = 73), whereas in the chloroplast dataset, it appears unresolved. A remarkable finding is that *Leucheria*, as presented by Jara-Arancio et al. (2017) and Katinas et al. (2022), appears as paraphyletic, as it includes *Polyachyrus* in both the nuclear and chloroplast datasets. *Leucheria* appears to be formed by three main clades, differing in many cases to the ones shown in Jara-Arancio et al. (2017). A first clade is formed by acaulescent species of *Leucheria*, coinciding with the clade “A” of Jara-Arancio et al. (2017). It includes *L.eriocephala*, *L.purpurea*, *L.achillaeifolia*, *L.hahnnii*, *L.leontopodioides*, *L.millefolium*, *L.candidissima*, *L.nutans*, *L.scrobiculata*, *L.cantillanensis*, and *L.salina*. This clade is strongly supported on both datasets (PP = 1.0, BS = 100). A second clade is formed by several species of *Leucheria*, plus *Polyachyrusfuscus*. It is moderately to strongly supported (PP = 0.99, BS = 64 for ITS; PP = 1.0, BS = 100 for chloroplast), and it is mostly comprised of tall perennials with leaves all along the stems, such as *L.bridgesii*, *L.lithospermifolia*, *L.rosea*, *L.garciana*, *L.gilliesii*, *L.meladensis*, *L.viscida*, and *L.polyclados*. This clade also includes annual species formerly recognized by Apodaca et al. (2021) as a single species, *Leucheriatomentosa*. Phylogenetic analyses show that samples formerly assigned to *L.tenuis*, *L.tomentosa*, *L.oligocephala*, *L.glandulosa*, *L.glabriuscula*, and *L.cerberoana* (currently synonymized with *L.tomentosa*) belong to five different clades. The nuclear dataset includes in this clade the morphologically distinct *L.floribunda*, whereas the chloroplast dataset is ambiguous regarding its position. A third clade retrieved with strong support (PP = 1.0, BS = 97 for ITS; PP = 1.0, BS = 94 for chloroplast) is mostly formed by perennials with a basal rosette, such as *L.hieracioides*, *L.integrifolia*, *L.runcinata*, *L.gayana*, *L.amoena*, *L.coerulescens*, and *L.glacialis*. It also includes two annual species considered by Katinas et al. (2022) as synonyms of *L.tomentosa*: *L.glandulosa* and *L.glabriuscula*. The two latter species form a strongly supported clade (PP = 1.0, BS = 97 for ITS; PP = 0.99, BS = 85 for chloroplast) with the putative new species, *L.peteroana*. In the combined analysis (Fig. 2), the *Marticorenia*, *Moscharia*, *Oxyphyllum*, *Polyachyrus* and *Leucheria* clade is again retrieved, with the same topology for *Marticorenia* and *Moscharia*. The clade comprised of *Oxyphyllum* + *Leucheria* + *Polyachyrus* is strongly supported (PP = 1.0, BS = 100), but the clade formed by *Leucheria* + *Polyachyrus* has low support (PP = 0.63, BS = 57). The three distinct clades of *Leucheria* are also retrieved. Finally, the phylogenetic position of *L.peteroana* is again confirmed as sister to the annual species *L.glandulosa* and *L.glabriuscula* with strong support (PP = 1.0, BS = 99).

### ﻿Taxonomic treatment

#### ﻿ Leucheria peteroana

Taxon classificationPlantaeAsteralesAsteraceae

Lavandero sp. nov.

1EFA9A81-221F-5D47-A937-FE8F2B86B936

urn:lsid:ipni.org:names:77351414-1

Figs 3 , 4

##### Diagnosis.

*Leucheriapeteroana* is most similar to *Leucheriaruncinata* but differs by its simple aboveground stems (vs. branching stems), solely glandular indumentum (vs. lanose and glandular), lack of any type of scent (vs. strongly pungent or fetid odour), completely white corollas on the adaxial side (vs. lilac to blue), pink-purplish anther apical appendages (vs. blue), pink style branches (vs. white styles), and pappus pectines of 250–520 µm long (vs. 130–160 µm) (Figs 5, 6). *Leucheriapeteroana* also differs from *Leucheriaapiifolia* by its larger height (vs. plants not taller than 30 cm.), two types of glandular trichomes in the vegetative part (vs. only one type of glandular trichome), lack of any type of scent (vs. soft and lemony odour), completely white corollas on the adaxial side (vs. pale gold corollas), outer lip completely extended at full anthesis (vs. outer lip revolute), and pink-purplish anther apical appendages (vs. beige to dark brown) (Fig. 7).

##### Type.

Chile • Región del Maule: Provincia de Curicó, comuna de Romeral. Lagunas de Teno, alrededores de la Laguna Planchón, sector Norte, 35°10'1.06"S, 70°32'37.79"W, 2593 m., 5 March 2019, fl. And fr., *N. Lavandero*, *L. Santilli y C. Ossa* 1873 (holotype: SGO 171859!; Isotype CONC!).

##### Description.

***Perennial*** herb 40–70 cm tall, forming clumps of seasonally persistent annual stems. ***Rhizome*** dark brown, round, 25–50 mm wide, bifurcating, oblique to vertical. ***Roots*** brown, ca. 1 mm wide. ***Stems*** green, erect, fistulose, 2.5–5.0 mm wide, simple, never branching, round, internodes up to 10 cm long, densely covered by two types of trichomes with clear and sticky resin, not fragrant, and without any pungent or noticeable scent when touched or pressed (same indumentum up to the corolla tube): short glandular, capitate, (60–)90–150 µm long, multicellular 8–15-celled trichomes; long glandular, (300–)500–1500(–2300) µm long, multicellular 10–30(–50)-celled trichomes. ***Leaves*** green, alternate; basal leaves attenuate, more densely arranged at the base, but not forming a conspicuous rosette; upper leaves sessile, amplexicaul, loosely arranged, gradually reduced in size towards the capitulescence. ***Lamina*** oblanceolate, pinnatipartite to pinnatisect, with 6–9 segments per side, almost tripartite towards the apex, (70–)130–180(–190) × (20–)50–60(–65) mm; base attenuate, amplexicaul, apex mucronate; margin serrate, texture coriaceous, densely glandulous on both surfaces; segments at the base 1(–2)-dentate, apex mucronate; segments in the middle 4–7-dentate; apical segments fused, 3–7-dentate; venation prominent on abaxial side, with primary vein ending in apical mucro, secondary veins ending in apical mucro of each segment, and tertiary veins ending in lateral teeth of each segment. ***Capitulescence*** a single corymbiform cyme per stem. ***Capitula*** 5–9 per stem, pedunculate, homogamous, discoid; pedicels (2–)8–10(–14) cm long. ***Involucres*** hemispheric 10.1–11.0 × 14.2–15.2 mm, two-seriate, alternate. ***Receptacle*** convex, epaleate (no flowers between bracts), glabrous. ***Outer involucral bracts*** (10–)12(–14), green, lanceolate, concave on the inner face, 8.1–9.2 × 1.8–1.9 mm, with 3 dark-green longitudinal veins (including the midrib), margin ciliate, apex ciliate, texture coriaceous to hyaline-membranaceous towards the margins, abaxial side densely covered by short and long glandular trichomes, adaxial side glabrous. ***Inner involucral bracts*** half the number of outer involucral bracts, (5–)6(–7), green, lanceolate, concave to flat 9.0–9.2 × 1.9–2.2 mm, with one dark-green longitudinal stripe (midrib), apex acute, texture leaf-like to hyaline-membranaceous towards both lateral margins, margin ciliate, central portion of the abaxial side sparsely covered by short glandular trichomes, hyaline lamina glabrous, adaxial side glabrous. ***Flowers*** isomorphic, bisexual, (40–)43(–45) per capitulum. ***Corollas*** bilabiate, white, sometimes pinkish white on the abaxial side, tube 4.3–4.6 mm long, 1.0–1.1 wide; corolla tube sparsely covered by glandular trichomes. ***Outer lip*** oblanceolate, 6.8–7.1 × 3.2–3.5 mm at its widest, apex 3-toothed, teeth equal, 4-veined, glabrous. ***Inner lip*** bifid, lacinae linear, 3.4–3.6 × 0.20–0.29 mm at its widest, connivent, glabrous. ***Stamens*** 5, 6.2–6.7 mm long, glabrous. ***Anthers*** sagittate, 3.0–3.3 mm long; apical appendages pink-purplish, lanceolate, 1.6–1.9 mm long, apex acute; tails long, lanceolate, 1.1–1.2 mm long, apex rounded, smooth. ***Style*** pink, 6.5–7.0 mm long, cleft into two truncate branches of pink colour, branches 1.1–1.3 mm long, with stigmatic papillae on internal surface and apical crown papillose. ***Cypselae*** dark-brown, 3.5–3.6 × 1.1–1.2 mm, obovoid, strigose; covered by two types of trichomes: glandular biseriate trichomes, 100–130 µm long, and twin trichomes, 230–280 µm long. ***Pappus*** uniseriate, fused at their bases into a ring, deciduous; bristles 23–30, white, sub-plumose, 7.4–7.7 mm long; pectines long, filiform, 250–400(–520) µm long, laterally inserted.

##### Distribution and habitat.

*Leucheriapeteroana* is endemic to the Andes of Central Chile. It is known only from the type locality on the whereabouts of Laguna El Planchón, Maule Region (Fig. 1). It grows at full sun in margins of Andean wetlands or shaded by large boulders and rock walls near 2500 m a.s.l. with SE orientation (Fig. 4). *L.peteroana* occurs associated with other high Andean plants such as *Grausalateritia* (Gillies ex Arn.) Weigend & R.H. Acuña, *Calceolariawilliamsii* Phil., *Acaenaovalifolia* Ruiz & Pav., *Calceolariafilicaulis* Clos, and *Erythranthelutea* (L.) G.L. Nesom.

##### Phenology.

Flowering between December and March. Fruiting in March.

##### Etymology.

The specific epithet refers to the active volcanic complex Planchón-Peteroa. The Andean Lake where the species occurs lies at the foot of this volcano.

**Figure 3.**
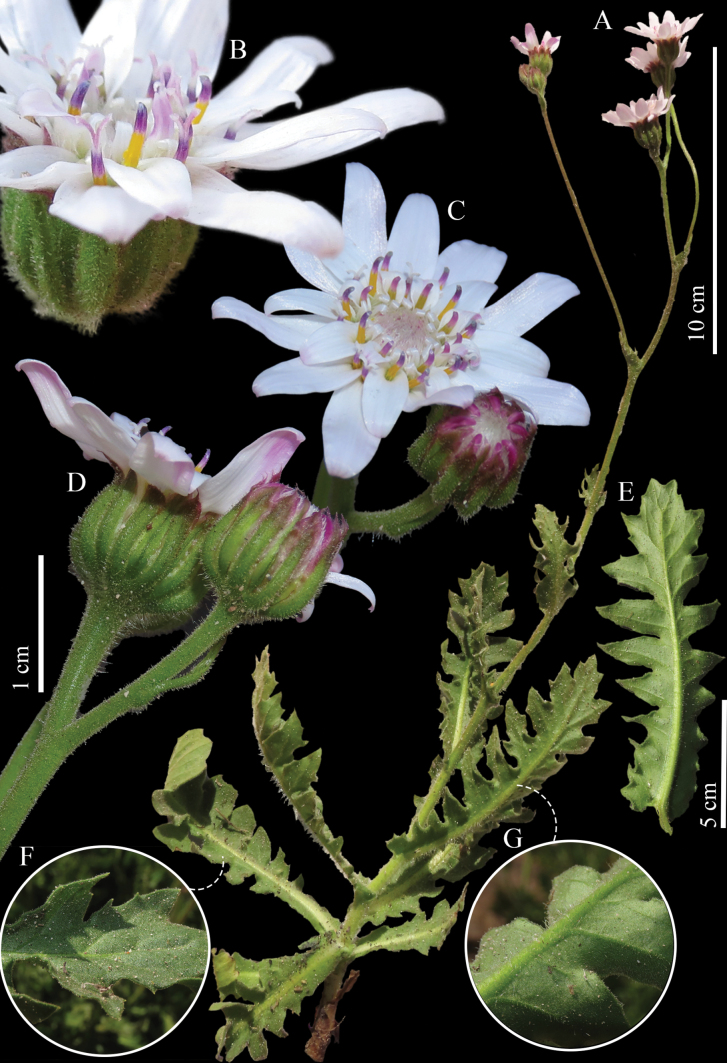
*Leucheriapeteroana* Lavandero, sp. nov. (Lavandero & Pérez 1504, SGO) **A** habit **B** detail of the capitulum, apical anther appendages and styles **C** capitulum, mid-upper view **D** capitulum, lateral view **E** leaf, abaxial side **F** leaf, detail of adaxial side **G** leaf, detail of abaxial side. All photographs by Nicolás Lavandero.

**Figure 4.**
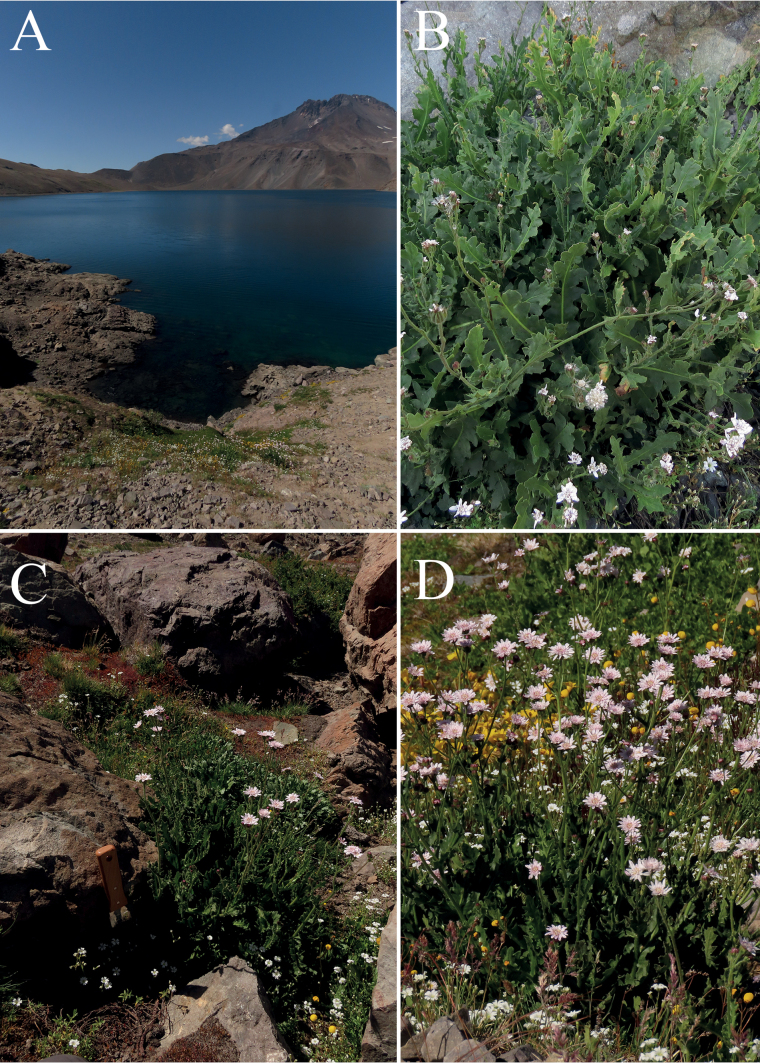
Habit and habitat of *Leucheriapeteroana* Lavandero, sp. nov. **A** overview of Laguna El Planchón and the volcanic complex Planchón-Peteroa **B** detail of the plant **C** another plant, growing among rocks **D** detail of plant clump at full sun exposure, but right next to an Andean spring. All photographs by Nicolás Lavandero.

##### Informal conservation status.

*Leucheriapeteroana* can be tentatively considered as Critically Endangered (CR) under the IUCN categories and criteria B2ab(ii,iii). Criterion B2 was selected because its Area of Occupancy is < 10 km^2^ (4 km^2^). Criterion “a” was selected because it is known to exist at only a single location, with only two known subpopulations. Criterion b(ii,iii) was selected because we expect a continuing decline of suitable conditions for the species to thrive. There is evidence of a decreasing snow cover extent during the dry season of near 15% per decade in the Andes at mid-latitudes (Cordero et al. 2019). It is also likely that explosive volcanic eruptions of the Planchón-Peteroa complex, close to the only known locality of the species, may wipe out the whole population. These events are relatively common, with at least 20 eruptions documented since 1600 CE, the most recent occurring between September 2018 and April 2019 (Romero et al. 2020). *Leucheriapeteroana* is not present in any known protected area. Although there have not been appropriate efforts to exhaustively locate more populations of *L.peteroana* in the area, it is likely that these would be subject to the same threats as the already known populations. The extent of occurrence (EOO) could not be calculated since only two populations are known.

**Figure 5.**
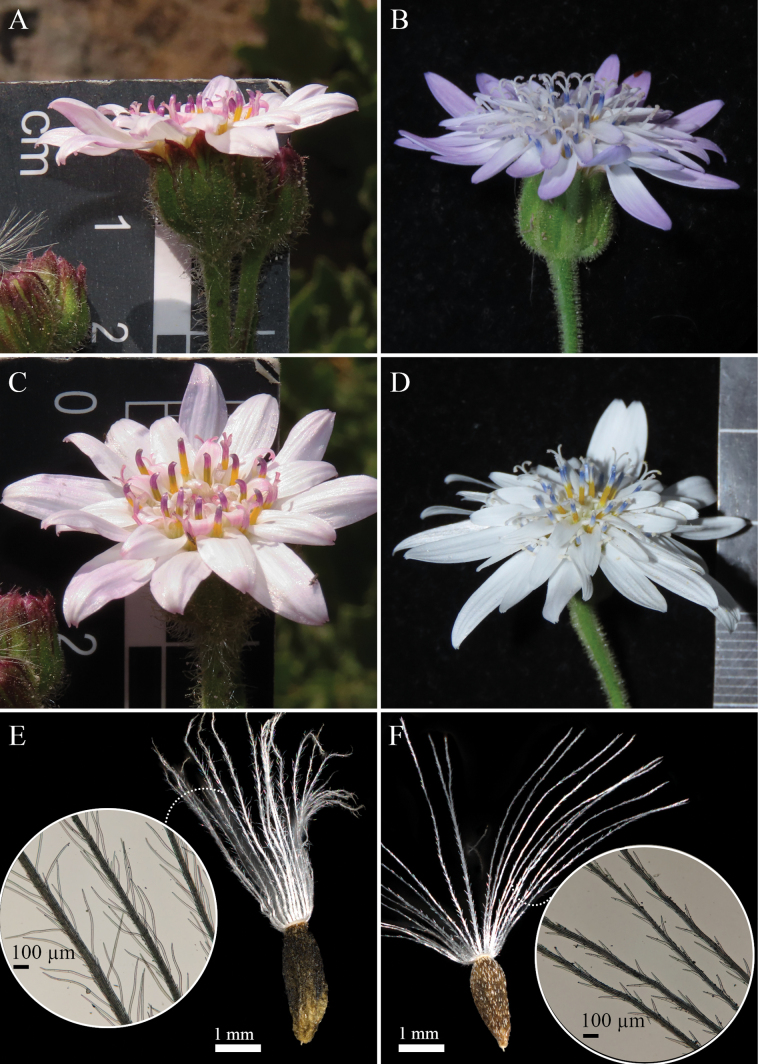
Comparison between *Leucheriapeteroana* (NL-1873, SGO) (**A, C, E**) and *Leucheriaruncinata* (NL-1867, SGO) (**B, D, F**) **A, B** frontal view of capitula **C, D** detail of capitula and flowers **E, F** cypselae and detail of pappus bristles. All photographs by Nicolás Lavandero, except E and F (Nicolás Pinilla).

##### Additional specimens examined.

Chile • Región del Maule: Provincia de Curicó, departamento de Curicó. A Orillas de la Laguna Teno. 2500 m. 10 March 1967. *Marticorena & Matthei 892* (CONC!); En los alrededores de la Laguna Teno. 2570 m. *Lavandero & Pérez 1504*. 8 January 2022 (SGO!).

##### Notes.

Crisci (1976) and posteriorly Katinas et al. (2022) identified *Marticorena & Matthei 892* as *Leucheriaapiifolia*. The differences between these species are notorious (Fig. 7), since the leaf shape and flower colour differ, but the fact that both plants lack lanate indumentum and the dark colour both species acquire once pressed, may have led to this misidentification.

**Figure 6.**
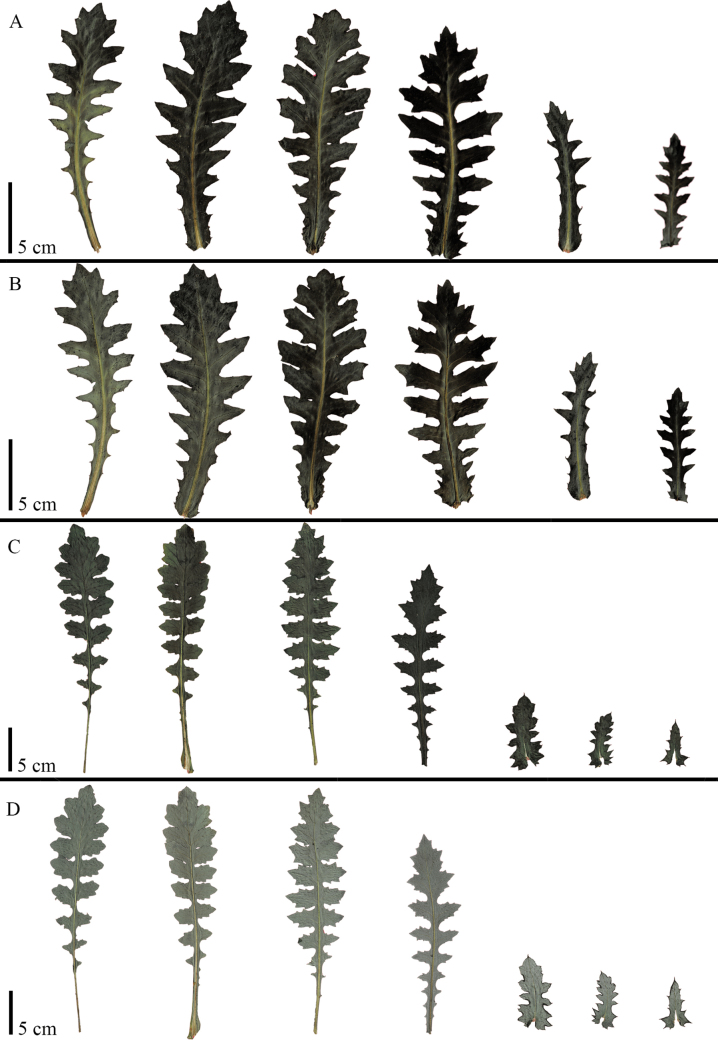
Leaf morphology comparison between *Leucheriapeteroana* and *Leucheriaruncinata*. For each leaf, both adaxial and abaxial sides are shown **A***Leucheriapeteroana*, adaxial side (NL 1873, SGO) **B***Leucheriapeteroana*, abaxial side (NL 1873, SGO) **C***Leucheriaruncinata*, adaxial side (NL-1867, SGO) **D***Leucheriaruncinata*, abaxial side (NL-1867, SGO).

**Figure 7.**
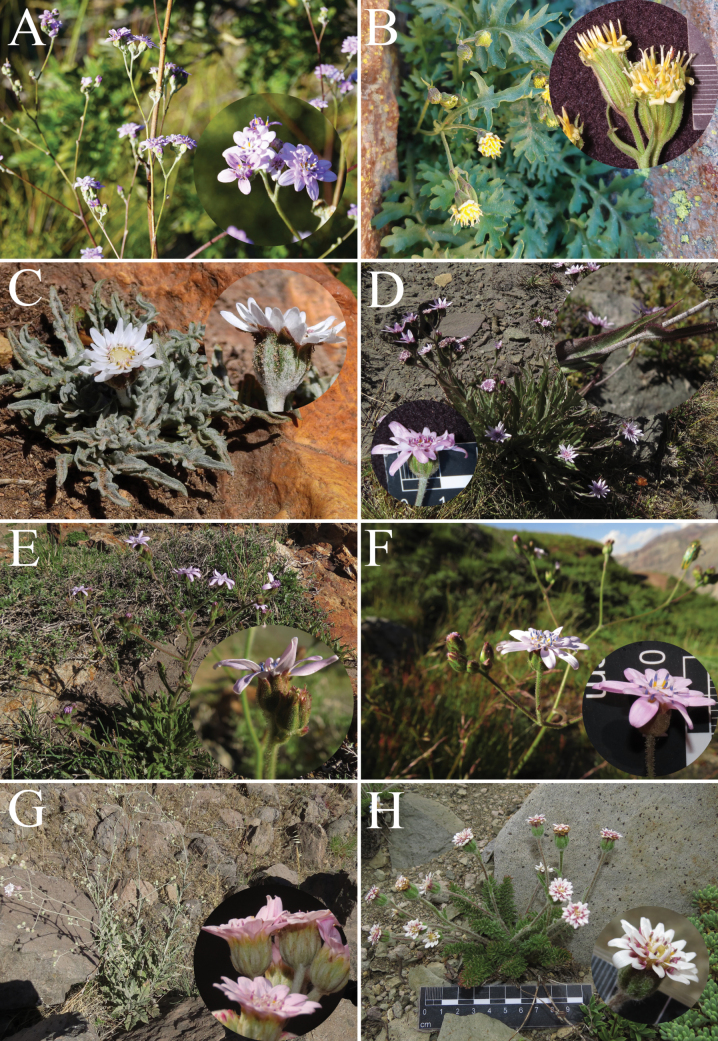
Other *Leucheria* species found along the Río Teno Basin in the Maule Region, Central Chile **A***Leucheriaamoena***B***Leucheriaapiifolia***C***Leucheriacandidissima***D***Leucheriagarciana***E***Leucheriaglacialis***F***Leucheriaintegrifolia***G***Leucherialithospermifolia***H***Leucheriamillefolium*. All photographs by Nicolás Lavandero, except *L.amoena* (Joaquín E. Sepúlveda) and *L.candidissima* (Guillermo Debandi).

### ﻿Key for the species of *Leucheria* present in the Río Teno basin

This key aims to cover the diversity of species collected by the authors around this valley. The taxonomy slightly differs from that of Katinas et al. (2022), as it recognizes *Leucheriagarciana* as a distinct taxonomic unit from *Leucheriagilliesii*, and *Leucheriamillefolium* from *Leucheriapurpurea*. These decisions are based on our own ongoing revision of the genus, based on field observations, phylogenetic analyses and herbarium work, rather than solely on the latter, as done by Katinas et al. (2022). Fresh material is often easy to identify. However, herbarium material can be challenging to identify, especially if the belowground structures are incomplete, and the colour of the flower structures are not recorded. Photographs of all species in the field, other than *L.peteroana*, are provided in Fig. 7.

**Table:** 

1	Plants acaulescent, not taller than 20 cm, with a long creeping rhizome with dark long roots emerging from the internodes	**2**
–	Plants caulescent, up to 170 cm, without a creeping rhizome with dark long roots emerging from the internodes, either with a basal leafy rosette or leaves distributed along the stem	**4**
2	Leaves with deeply appressed and dense lanate indumentum in both faces, greyish in appearance	***L.candidissima*** (Fig. 7C)
–	Leaf indumentum not as above, green in appearance	**3**
3	Leaves with glandular indumentum only, citric pungent scent when crushed. Flowers yellow	***L.apiifolia*** (Fig. 7B)
–	Leaves with sparsely lanate indumentum, without any scent. Flowers pink	***L.millefolium*** (Fig. 7H)
4	Plants with a conspicuous basal rosette or leaves distributed mostly at the basal portion of the stem	**5**
–	Plants with leaves distributed along the stem	**8**
5	Plants with a combination of glandular and lanate indumentum. Flowers lilac to pink, anther apical appendages blue, styles white	**6**
–	Plants with only glandular indumentum along the whole plant, flowers white, anther apical appendages pink, styles pink	***L.peteroana*** (Figs 3, 4, 5, 6)
6	Plants up to 1.7 m tall, with a dominantly lanate indumentum at leaves and stem, glandular indumentum appears in concomitance with lanate indumentum at the distal part of capitulescence. Plants non-sticky	**7**
–	Plants less than 50 cm tall, with a dominantly glandular indumentum on the stem and capitula, with trichomes arising perpendicular to the stem and well above any lanate indumentum. Leaves with lanate indumentum at abaxial side. Plants sticky when touched	***L.glacialis*** (Fig. 7E)
7	Plants with capitula densely arranged at the distal part or capitulescences. Involucre densely tomentose	***L.amoena*** (Fig. 7A)
–	Plants with capitula evenly arranged along the capitulescence. Involucre with glandular and lanose indumentum	***L.integrifolia*** (Fig. 7F)
8	Plants with capitate glandular indumentum emerging well above the appressed lanate indumentum in most organs, involucre mostly glandular, flowers pink	***L.garciana*** (Fig. 7D)
–	Plants with mostly appressed lanate indumentum in all organs, involucre lanate, flowers white to lilac.	***L.lithospermifolia*** (Fig. 7G)

## ﻿Discussion

Chloroplast markers and the nuclear ribosomal cistron regions have been extensively used to infer phylogenetic relationships within Nassauvieae (Katinas et al. 2008c; Jara-Arancio et al. 2017; Jara-Arancio et al. 2018; Sancho et al. 2018; Nicola et al. 2019). However, not all studies documented the topological incongruences between the chloroplast and nuclear ribosomal cistron. Moreover, only a few of these studies explicitly performed tests to evaluate incongruence between nuclear and chloroplast partitions. In the present study, we have confirmed that there are several topological incongruences between these two datasets for Nassauvieae at both generic and infrageneric level. These results suggest that any systematic study and reclassification proposal within Nassauvieae using these datasets alone or combined should be taken into consideration with caution.

In the present study, *Leucheria*, as recognized by Crisci (1976) and more recently by Katinas et al. (2022), appears to be paraphyletic, including the morphologically distinct genus *Polyachyrus*. This result was not shown by Jara-Arancio et al. (2017), as it did not include any sample of *Polyachyrus*. Additionally, the position of *Oxyphyllum* as sister to *Leucheria* + *Polyachyrus* clade was not retrieved by Jara-Arancio et al. (2017), as it also did not include *Oxyphyllum* in the sampling. Our analyses, including 25 of the 27 accepted genera of Nassauvieae, consistently retrieved a clade comprising *Leucheria*, *Moscharia*, *Marticorenia*, *Oxyphyllum* and *Polyachyrus*.

Within *Leucheria*, an interesting finding is that our phylogenetic results are more consistent with the taxonomy proposed by Crisci (1976) than the recent proposal by Katinas et al. (2022), which dramatically reduced the number of accepted species of *Leucheria*. The synonymization of *Leucheriamillefolium* into *Leucheriapurpurea* is not supported by our phylogenetic analyses. Likewise, the synonymization of *Leucheriagarciana* into *Leucheriagilliesii* is also not supported by the present work. The proposal of lumping 10 species of *Leucheria* into *Leucheriatomentosa* by Apodaca et al. (2021), including all the former annual species recognized by Crisci (1976) is here contested. Based on morphology and the revision and comparison of our own field collections with the type specimens, we sampled at least six taxa recognized as annuals by Crisci (1976), and lumped into *Leucheriatomentosa* by Apodaca et al. (2021). Interestingly, our phylogenetic results indicate that *Leucheriatomentosa*, as recognized by Katinas et al. (2022), is not monophyletic, suggesting that an important taxonomic work on this group needs to be done, considering both field, herbarium and phylogenetic evidence available (Lavandero et al., in prep.). An unexpected finding was that the new species *Leucheriapeteroana*, is sister to a clade comprised by two annual species found in the Mediterranean region of Central Chile, *Leucheriaglandulosa* and the poorly collected *Leucheriaglabriuscula*. Although these three species have little in common morphologically, it is noteworthy that *Leucheriaglandulosa* has pink anther appendages, whereas the sister clade comprises species with only blue anther appendages. This character, although rarely recorded on herbarium sheets, is taxonomically important. Likewise, *Leucheriaglabriuscula* has a similar pappus, with long pectines (~300–400 µm long), an unusual character within the genus.

The discovery of a new species of *Leucheria*, restricted to a small region of the Andes of Central Chile highlights the importance of more field sampling, even for widely collected genera. Most herbarium collections, including those in Chile, have a significant collection bias (Daru et al. 2017; Hughes et al. 2021), particularly noticeable in areas with harsh topography and lack of roads, as most of the Andes Mountain range. The access to the Lagunas del Teno, and therefore, the finding of *Leucheriapeteroana*, was only possible because there is an unpaved road that leads to a dam that controls the water flow from the lake, used for irrigation (Caputo et al. 2013). This area is also known to harbour several rarely collected species, such as *Calceolariawilliamsii* Phil. and *Leucheriaapiifolia*. As more fieldwork is done in remote areas of the Andes, it is more likely that new species will be found, particularly those with restricted distributions or with very specific requirements, as confirmed by recent additions to the Andean flora of Chile (Villarroel et al. 2021, 2022; Menegoz et al. 2024).

This work highlights the importance of taxonomic revisions that integrate herbarium studies, field collections and ecology, along with a robust phylogenetic framework, especially for diverse and complex genera, such as *Leucheria*. Revisions made solely on herbarium species may leave behind important characters that are only visible when dealing with live plants in the field, such as life form, growth habit, texture, odours and colours that eventually fade once pressed. A fully resolved phylogeny of *Leucheria* is still lacking, and its monophyly has been put to the test by our results. In future works, we will aim to resolve the systematics of Nassauvieae, particularly *Leucheria* and closely related genera, based on low-copy nuclear genes, following a target capture approach (Mandel et al. 2014).

## Supplementary Material

XML Treatment for Leucheria peteroana

## ﻿Additional information

### Author ORCIDs

Nicolás Lavandero https://orcid.org/0000-0002-2390-5078

Fernanda Pérez https://orcid.org/0000-0001-5730-9760

Nicolás Pinilla https://orcid.org/0000-0002-1494-215X

### Data availability

All of the data that support the findings of this study are available in the main text or Supplementary Information.

## Supplementary materials

Supplementary material 1 Phylogenies of Nassauvieae resulting from Maximum likelihood of nuclear ITS, and chloroplast *rpl32-trnL* and *trnL-trnF* datasets Data type tif https://binary.pensoft.net/file/1169965This dataset is made available under the Open Database License (http://opendatacommons.org/licenses/odbl/1.0/). The Open Database License (ODbL) is a license agreement intended to allow users to freely share, modify, and use this Dataset while maintaining this same freedom for others, provided that the original source and author(s) are credited.Author: Nicolás Lavandero

Supplementary material 2 Phylogenies of Nassauvieae resulting from Bayesian analyses of nuclear ITS, and chloroplast *rpl32-trnL* and *trnL-trnF* datasets Data type tif https://binary.pensoft.net/file/1169966This dataset is made available under the Open Database License (http://opendatacommons.org/licenses/odbl/1.0/). The Open Database License (ODbL) is a license agreement intended to allow users to freely share, modify, and use this Dataset while maintaining this same freedom for others, provided that the original source and author(s) are credited.Author: Nicolás Lavandero

Supplementary material 3 GenBank accession numbers for the ITS, *rpl32-trnF*, and *trnL-trnF* sequences used in this study Data type xlsx https://binary.pensoft.net/file/1169967This dataset is made available under the Open Database License (http://opendatacommons.org/licenses/odbl/1.0/). The Open Database License (ODbL) is a license agreement intended to allow users to freely share, modify, and use this Dataset while maintaining this same freedom for others, provided that the original source and author(s) are credited.Author: Nicolás Lavandero
