# Supplementary figures and images for: ﻿Leucheriapeteroana (Nassauvieae, Asteraceae), a new species of Leucheria endemic to the Andes of Central Chile, and insights into the systematics of Nassauviae
Source: PhytoKeys. 2024 Nov 7;248:315–37. doi: 10.3897/phytokeys.248.133202 (PMC11565183; doi:10.3897/phytokeys.248.133202)

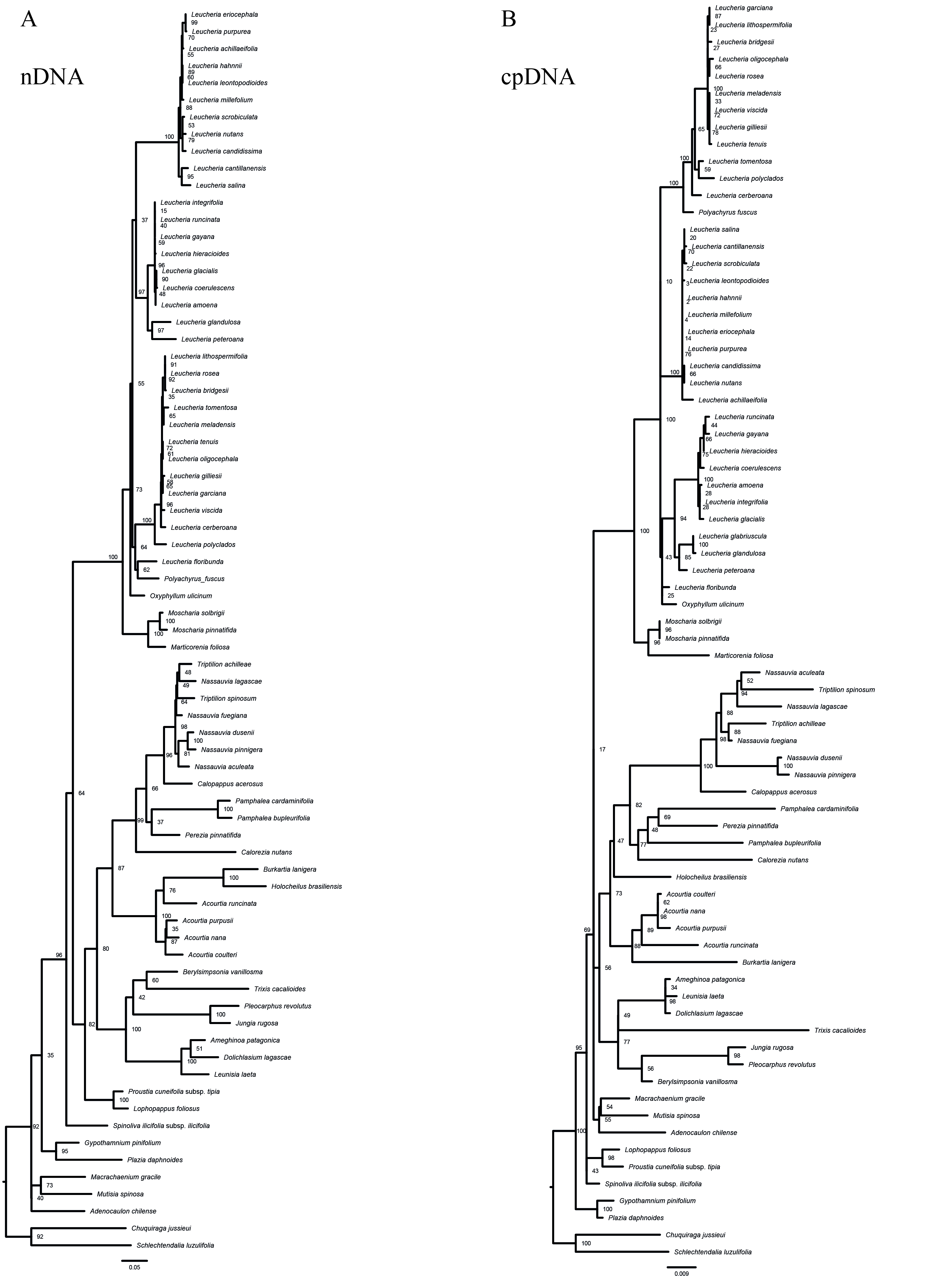

Supplement: Supplementary material 1 — Phylogenies of Nassauvieae resulting from Maximum likelihood of nuclear ITS, and chloroplast rpl32-trnL and trnL-trnF datasets [file phytokeys-248-315_article-133202__-s001.tif]

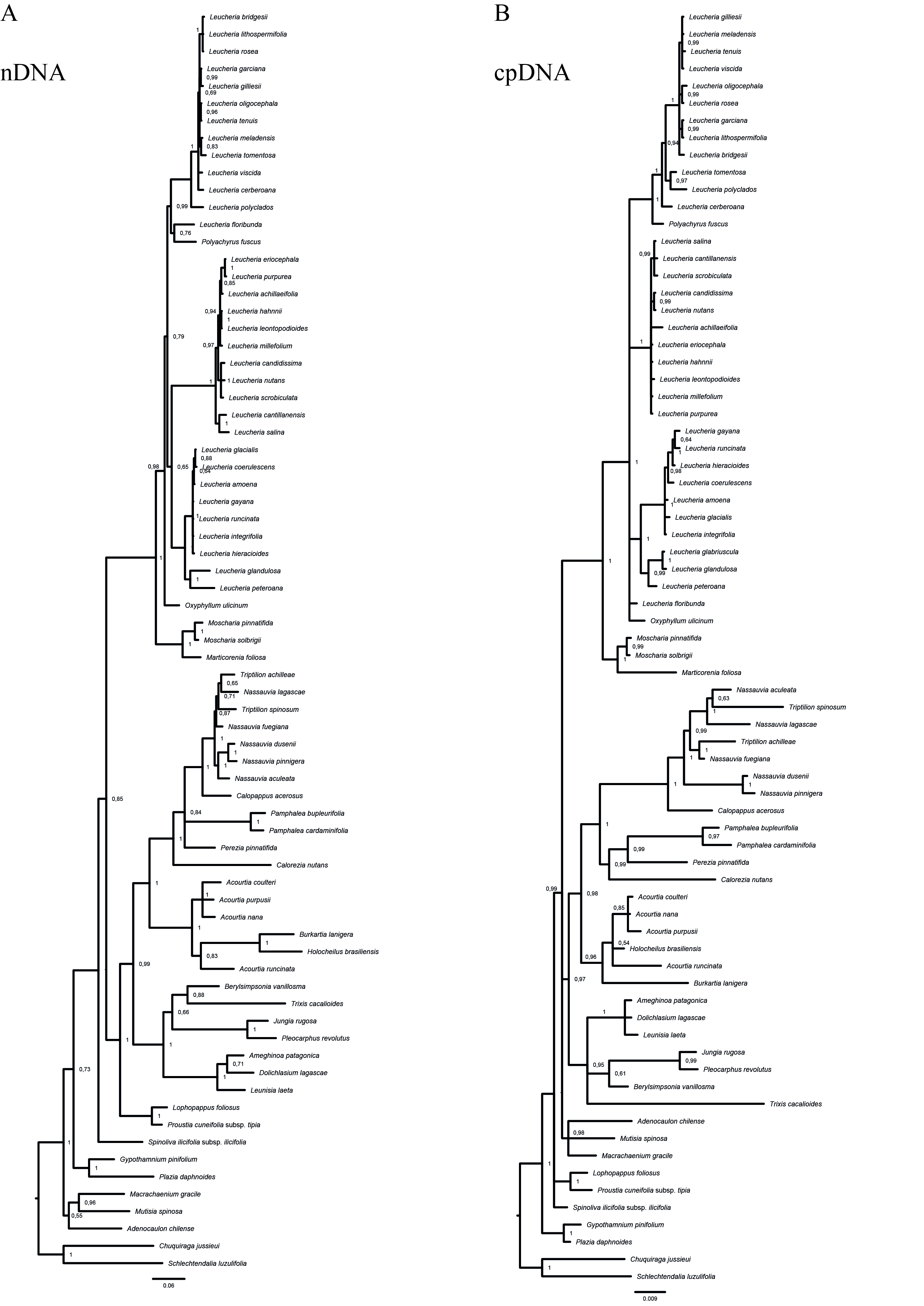

Supplement: Supplementary material 2 — Phylogenies of Nassauvieae resulting from Bayesian analyses of nuclear ITS, and chloroplast rpl32-trnL and trnL-trnF datasets [file phytokeys-248-315_article-133202__-s002.tif]
